# Advancement of Protein- and Polysaccharide-Based Biopolymers for Anthocyanin Encapsulation

**DOI:** 10.3389/fnut.2022.938829

**Published:** 2022-06-17

**Authors:** Jiahui Song, Yue Yu, Minghuang Chen, Zhongyang Ren, Lin Chen, Caili Fu, Zheng feei Ma, Zhanming Li

**Affiliations:** ^1^School of Grain Science and Technology, Jiangsu University of Science and Technology, Zhenjiang, China; ^2^National University of Singapore Suzhou Research Institute, Suzhou, China; ^3^College of Ocean Food and Biological Engineering, Jimei University, Xiamen, China; ^4^Food, Nutrition and Health, Faculty of Land and Food Systems, The University of British Columbia, Vancouver, BC, Canada; ^5^Department of Health and Environmental Sciences, Xi'an Jiaotong-Liverpool University, Suzhou, China

**Keywords:** anthocyanins, biopolymer, polysaccharides, encapsulation, stability, bioavailability

## Abstract

Although evidence shows that anthocyanins present promising health benefits, their poor stability still limits their applications in the food industry. Increasing the stability of anthocyanins is necessary to promote their absorption and metabolism and improve their health benefits. Numerous encapsulation approaches have been developed for the targeted release of anthocyanins to retain their bioactivities and ameliorate their unsatisfactory stability. Generally, choosing suitable edible encapsulation materials based on biopolymers is important in achieving the expected goals. This paper presented an ambitious task of summarizing the current understanding and challenges of biopolymer-based anthocyanin encapsulation in detail. The food-grade edible microencapsulation materials, especially for proteins and polysaccharides, should be employed to improve the stability of anthocyanins for effective application in the food industry. The influence factors involved in anthocyanin stability were systematically reviewed and highlighted. Food-grade proteins, especially whey protein, caseinate, gelatin, and soy protein, are attractive in the food industry for encapsulation owing to the improvement of stability and their health benefits. Polysaccharides, such as starch, pectin, chitosan, cellulose, mucilages, and their derivatives, are used as encapsulation materials because of their satisfactory biocompatibility and biodegradability. Moreover, the challenges and perspectives for the application of anthocyanins in food products were presented based on current knowledge. The proposed perspective can provide new insights into the amelioration of anthocyanin bioavailability by edible biopolymer encapsulation.

## Highlights

- The interactions between food matrix and anthocyanins were discussed in detail.
- The influence factors involved in the stability of anthocyanins were introduced.
- Performance of proteins or/and polysaccharides-based encapsulation was concluded.
- Advantages of protein-polysaccharide systems for encapsulation were summarized.

## Introduction

Recently, numerous studies have updated the current understanding of the health-promoting effects of dietary polyphenols and related food products (1–3). As an important and well-considered type of polyphenol, non-toxic water-soluble anthocyanins contribute to food color and present a wide range of biological activities, including antibacterial, anti-inflammatory, anti-diabetic, anti-obesity, and anticancer effects (4–6). However, the low stability and non-targeted release of anthocyanins have become the main obstacles in realizing their biological benefits in food systems (7, 8).

The main challenges for the application of anthocyanins in the food industry are how to decrease anthocyanin loss and control anthocyanin reaction to obtain more products with high stability (9, 10). Encapsulation systems can introduce physical protection for anthocyanins to achieve the stimulus-responsive controlled release and site-specific delivery of anthocyanins (11, 12). Many encapsulation methods have been performed for the controlled release of anthocyanins to overcome the poor stability, oral bioavailability, and intestinal absorption of anthocyanins.

In addition to delivery techniques or carriers, various cross-linked biopolymers have also been studied for anthocyanin encapsulation (13, 14). Suitable encapsulation materials are important for achieving the expected performance of anthocyanins. Undoubtedly, only edible materials can be developed for the delivery of anthocyanins in food applications (10, 15). Edible biopolymer-based systems, including proteins and carbohydrates, are preferred for anthocyanin encapsulation (16, 17).

Encapsulated systems based on protein or/and polysaccharide particles can protect anthocyanins in food products during storage and retain the bioavailability of anthocyanins within the gastrointestinal tract (18, 19). In this perspective, the current understanding of biopolymer-based anthocyanin encapsulation is presented in this paper in detail. The influence factors involved in anthocyanin stability are introduced, and the properties and performances of anthocyanins encapsulated by proteins or/and polysaccharide-based systems are summarized in detail. Moreover, the challenges and future perspectives of the application of anthocyanins in food products are highlighted. Retaining the bioavailability of anthocyanins by means of edible biopolymers encapsulation can provide much information for the promising application of anthocyanins in food products.

## Stability of Anthocyanins

### Factors Affecting the Stability of Anthocyanins

**Figure 1:**
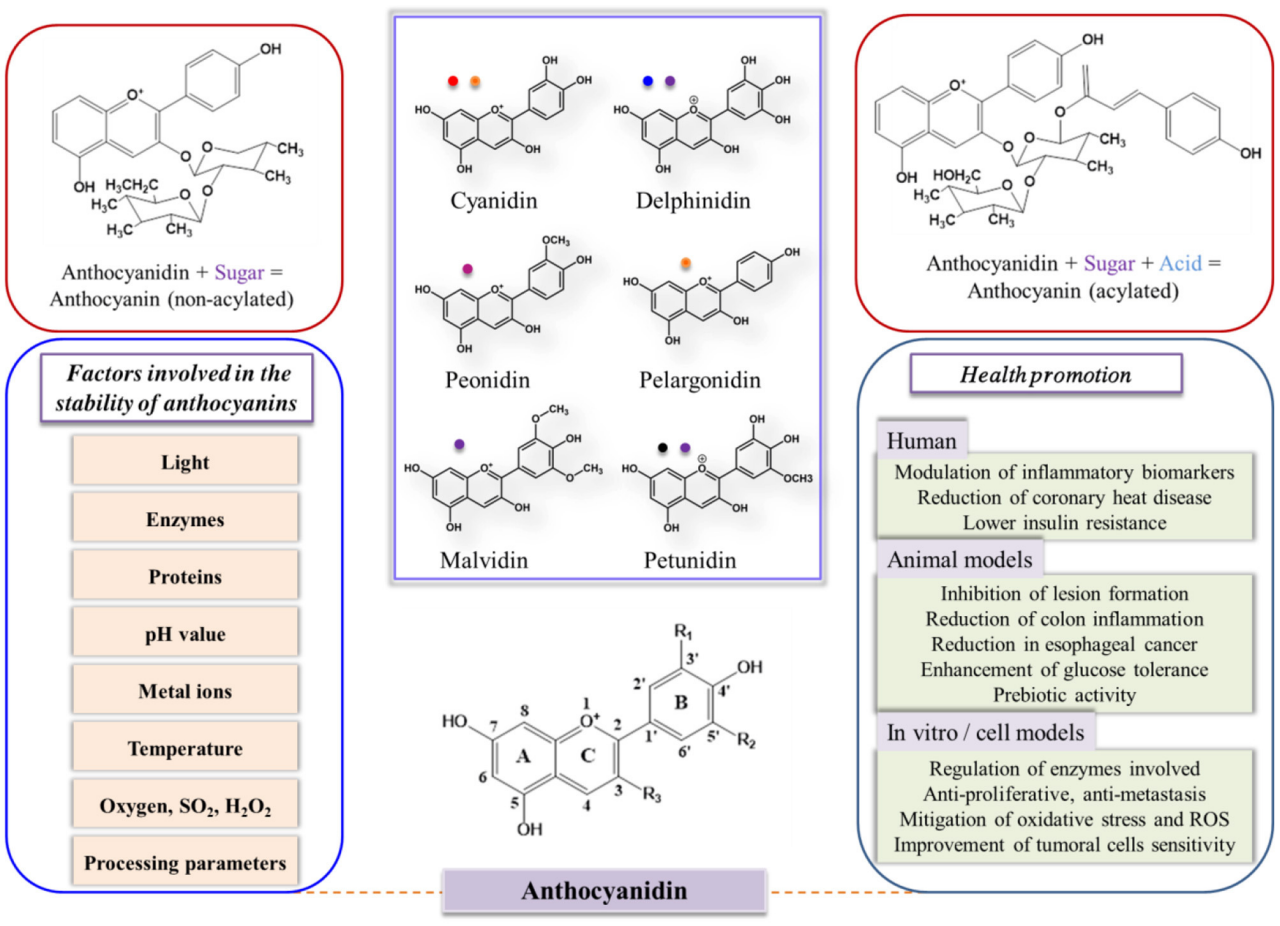
Structures, main colors involved, and bioactivity of six important food anthocyanidins, as well as factors affecting the stability of anthocyanins. *R*_1_*, R*_2_ = H or OH; *R*_3_ = H or glucose. The parameters were adapted from (12, 21, 23–27) with permission.

Anthocyanins have a carbon skeleton made up of C6–C3–C6 unit (xanthine cation) and are composed of anthocyanidin (aglycone units) linked to sugar, which is usually located at the 3-position on the C-ring and methoxyl and hydroxyl groups (20), as shown in Figure 1. However, the stability of anthocyanins is strongly related to the substitution pattern in the B-ring; the stability can be improved with the increase in methoxyl group or deteriorate with the increase in hydroxyl group. Glycosylation and acylation can improve the stability of anthocyanins (21, 22).

In general, the application of anthocyanins as food additives is seriously limited by their instability. The absorption of anthocyanins is small in comparison with the dietary consumption of anthocyanins, indicating the low bioavailability of anthocyanins (28, 29). Anthocyanins may easily be degraded *in vivo* before reaching the target locations because of the harsh environment. As shown in Figure 1, anthocyanin stability can be easily impacted by pH, structure, enzymes, light, temperature, oxygen, solvents, concentrations, and other compounds that can interact with anthocyanins (12, 24). All of these factors restrict the wide applications of anthocyanins, because anthocyanins are extremely unstable and can easily degrade. Hence, the industrialized applications of anthocyanins in food products are challenging.

### Edible Encapsulation Materials

To date, although evidence shows that anthocyanins present promising health benefits, their poor stability still limits their applications in food industry. Foods containing anthocyanins can only enter the bloodstream for further absorption and metabolism after reaching the gut lumen (30). Therefore, increasing the stability of anthocyanins is necessary to promote their absorption and metabolism and improve their health benefits. Encapsulated delivery systems have been reported to protect anthocyanins from adverse environmental conditions (31–34).

**Figure 2:**
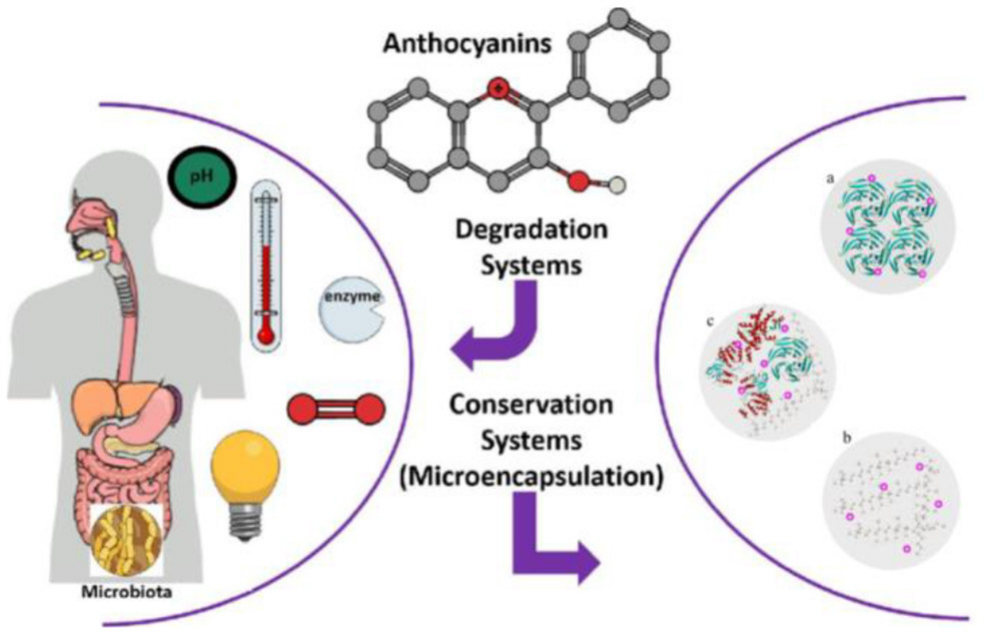
Schematics of anthocyanin degradation and biopolymer-based microencapsulation systems (a-protein particles, b-polysaccharide particles, and c-protein *plus* polysaccharide particles). Reprinted or adapted from references (12, 32–34) with permission.

Although several wall materials can be employed for encapsulation, some properties, such as affinity, film-forming ability, degradability, intestinal resistance, and viscosity, should be optimized before the selection of wall materials (23, 35). Edible wall materials can be made from gum, protein, polysaccharides (natural or modified), and synthetic polymers (36, 37). The food-grade proteins and polysaccharides that are generally recognized as suitable materials for food products are shown in Table 1. Therefore, edible microencapsulation materials, especially for proteins and polysaccharides, should be clarified to improve the stability of anthocyanins for effective application in the food industry (Figure 2).

**Table 1:** Properties of encapsulation materials for anthocyanins.

**Source of anthocyanins**	**Encapsulation materials**	**Properties**	**References**
Extract from jaboticaba pomace	Maltodextrin, pectin, and soy protein isolate	Decreases the degradation caused by UV radiation	(38)
Black soybean seed coat extracts	Soy protein isolate	Decreases the degradation rate and improves stability	(39)
Powdered BRS violeta grape juice	Soy protein and whey protein	Increases stability for long shelf life	(40)
Elderberry (*Sambucus nigr*a L.)	Whey protein and pectin	Increases encapsulation efficiency	(41)
*Camelina sativa* L. *Crantz*	Neutral polysaccharides and proteins	Increases stability	(42)
Black currant extract	Whey protein isolate, inulin, and chitosan	Increases stability	(43)
Sweet cherry skins	Whey proteins	Increases stability	(44)
Blueberry	Whey protein isolate	Improves bioactivity	(45)

## Protein- and Polysaccharide-Based Encapsulation

### Protein-Based Encapsulation

Food-grade proteins, especially whey protein, caseinate, gelatin, and soy protein, are attractive in the food industry owing to their health benefits. Their functional properties, including gelation, emulsification, and binding capacity, support their use as alternatives in the development of anthocyanin delivery systems (46, 47). In addition, proteins' hydrophobic region can interact with the benzene ring of anthocyanins. The carbonyl and amine groups of proteins form hydrogen bonds with the hydrophilic region of anthocyanins (48).

Whey proteins could be developed as wall materials to deliver anthocyanins with enhanced bioavailability (21, 35). Whey protein microgels as an anthocyanin encapsulation material can dissolve rapidly in the gastrointestinal tract and form liquid particles that impede anthocyanin release and degradation (49). The interactions between whey proteins and anthocyanins affect the color and heat/light stability of anthocyanins. The encapsulation of anthocyanins from blackcurrant using whey protein via spray drying or freeze drying has been suggested to develop nutritional food products (50). The encapsulation of anthocyanins from sour cherry skins using whey proteins with suitable encapsulation efficiency (over 70%) decreases gastric digestion and thus presents a potential as a functional matrix for food products (51).

**Figure 3:**
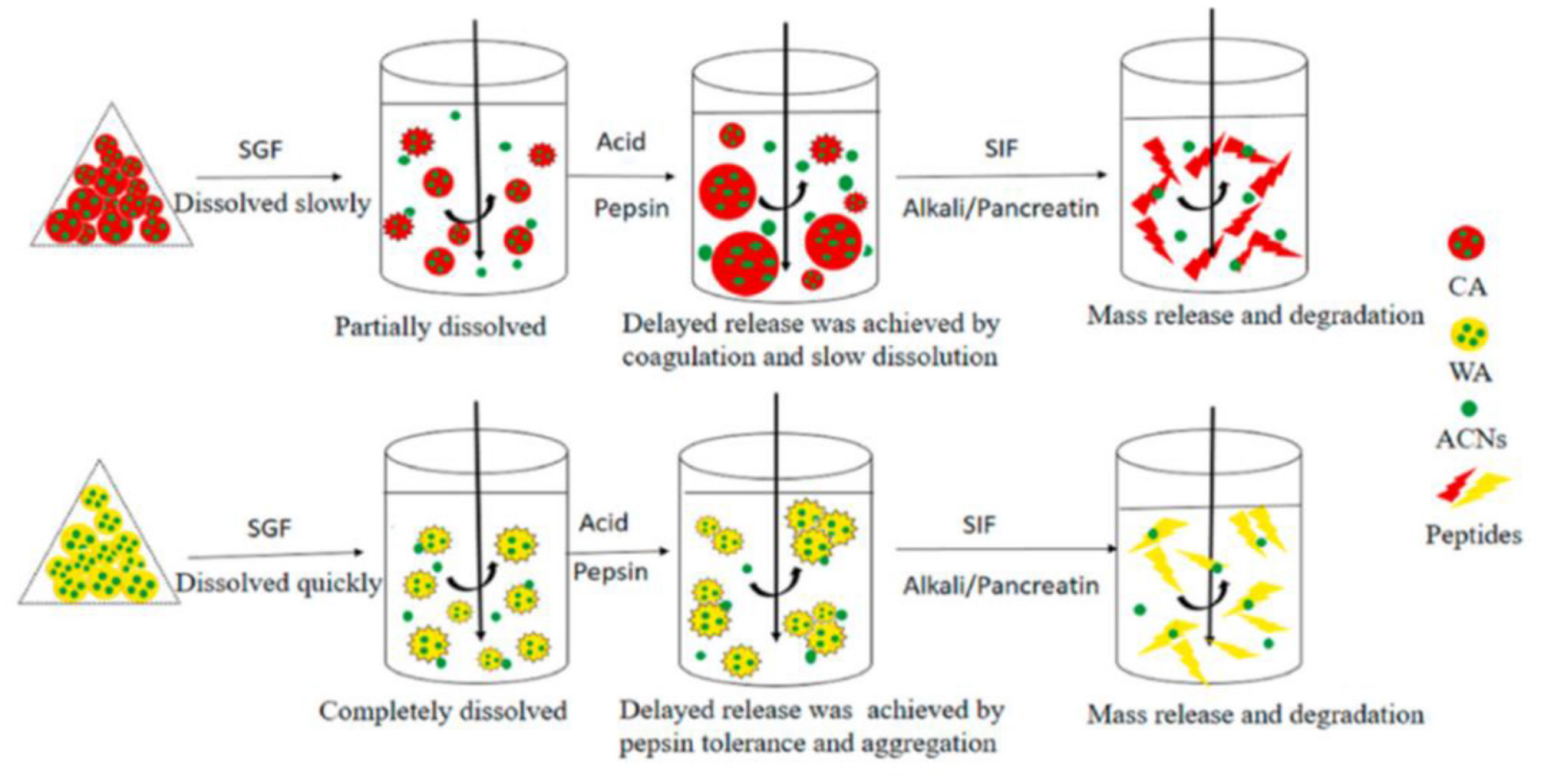
Release mechanisms of CA-ACN and WA-ACN microparticles during *in vitro* digestion. Reproduced from reference (36) with permission. CA, casein; WA, Whey protein isolate; ACN, anthocyanin; SGF, simulated gastric fluid; SIF, simulated intestinal fluid.

**Figure 4:**
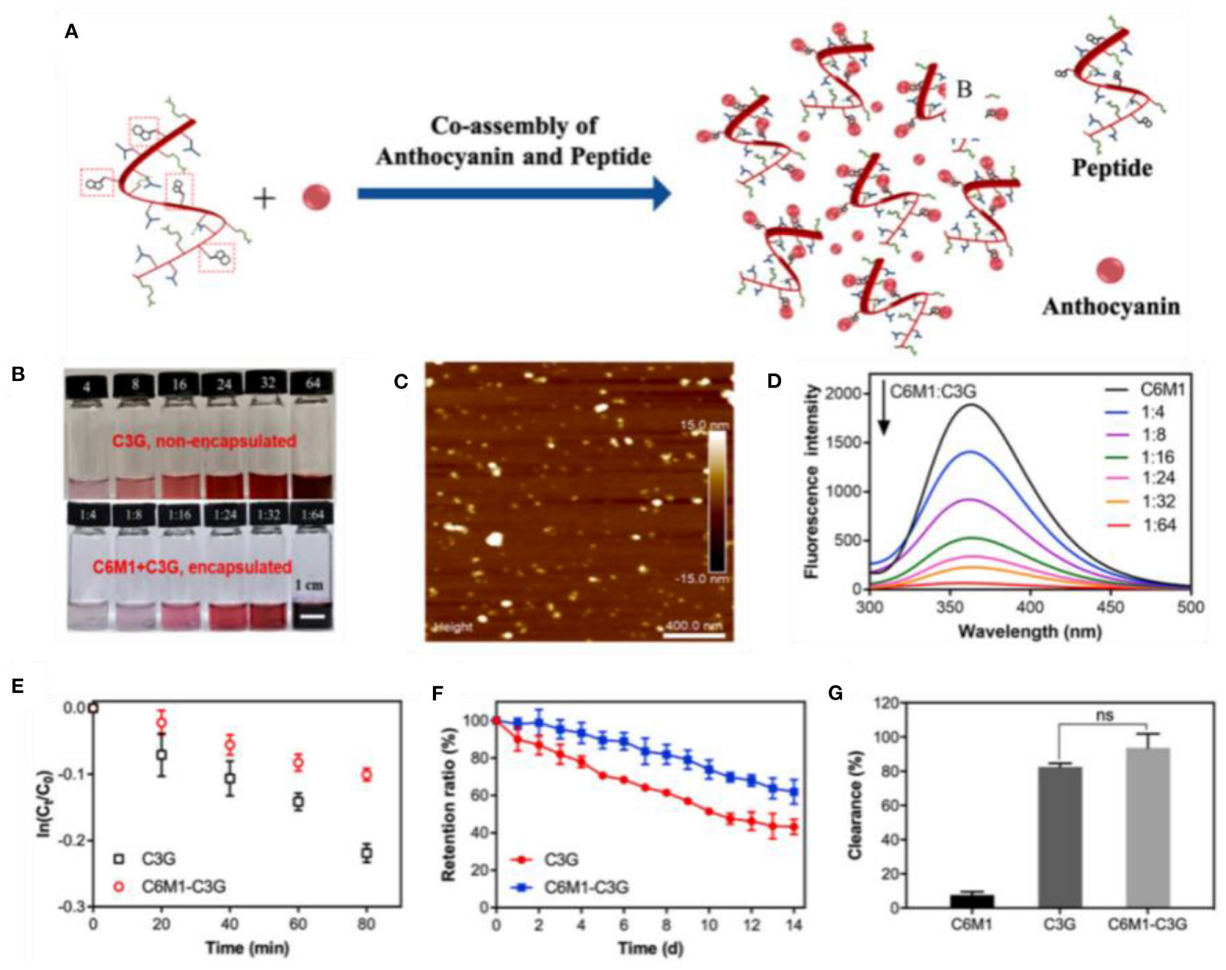
**(A)** Schematic of the co-assembly of the peptide (C6M1) and anthocyanin (C3G) into a nanocomposite. **(B)** Encapsulated and non-encapsulated anthocyanins. **(C)** Atomic force microscopy image and **(D)** fluorescence spectra of C6M1–C3G nanocomposites. **(E)** Thermal stability (80 °C), **(F)** retention ratio during storage (25 °C), and **(G)** activity tests of C3G and C6M1–C3G. Reproduced from reference (52) with permission.

Whey protein, casein, and soy protein isolates are efficient for improving anthocyanin bioavailability (Table 1). Casein and whey protein have been used as wall materials to encapsulate blueberry anthocyanins using spray drying technique. Anthocyanin encapsulation is helpful in decreasing the rapid release and degradation of anthocyanin, especially during digestion in simulated gastric fluid. However, casein and whey protein showed different protection mechanisms as shown in Figure 3. The formation of casein–anthocyanin microparticles with poor solubility effectively inhibited the release and degradation of anthocyanins. The highly soluble whey protein–anthocyanin microparticles had decreased anthocyanin release. Casein and whey protein isolate could be employed to hinder the release of encapsulated anthocyanins, indicating that the proteins' physicochemical properties and structural changes caused by digestion contributed to anthocyanin delivery. Obviously, the individual digestion behaviors of different proteins or composites as wall materials for anthocyanin encapsulation should be investigated in future research. The conformational change of the amphiphilic peptides of 18 amino acids (C6M1) from an α-helical structure to a β-sheet structure was caused by co-assembly when used for anthocyanin encapsulation (Figure 4). The C6M1 peptide improved the resistance of anthocyanin to pH, high temperature, and metallic ions and improved the bioactivity for scavenging free radicals (52).

Although anthocyanin–protein interactions have been extensively studied, many parameters still need to be evaluated (53). The chemical structures of anthocyanins contribute to binding affinity. Moreover, different anthocyanins may produce different binding forces with proteins; hence, binding affinity to specific anthocyanins should be explored (54). The protein concentrations used in combination with anthocyanins still need to be optimized because they influence the rheological and sensory properties of anthocyanin–protein complexes, which are crucial parameters for food and beverage products (21).

### Polysaccharide-Based Encapsulation

Polysaccharides, such as starch, pectin, chitosan, cellulose, mucilages, and their derivatives, are used as encapsulation materials because of their satisfactory biocompatibility and biodegradability (55). The performances of starch and its derivatives have been evaluated for anthocyanin encapsulation. Non-toxic and biodegradable chitosan has been widely utilized for anthocyanin encapsulation. Anthocyanin–chitosan nanoparticles are formed via non-covalent bonds (e.g., weak ionic binding and hydrogen binding) (56). As reported, dual coating with chitosan and polyanionic polysaccharide to stabilize anthocyanins had high encapsulation efficacy and achieved resistance against auto-oxidation, heat, ascorbic acid, and neutral environment (57).

In addition, as the most widely reported cyclic oligosaccharide material, cyclodextrin can form complexes with anthocyanins through hydrogen bonding and hydrophobic interactions (58). Maltodextrin is also commonly introduced in the food industry as a wall material. The dextrose equivalent of maltodextrin is of paramount importance for retaining the stability and other properties of anthocyanins (59). Short-chain maltodextrin with high dextrose equivalent resulted in browning, hygroscopicity, and solubility. However, maltodextrin with s a higher dextrose equivalent showed better performance in retarding anthocyanin degradation (60, 61).

The combination of xanthan gum and carboxymethyl starch produced a high encapsulation efficiency (over 96%) and contributed to the stability of blueberry anthocyanins (62). The co-encapsulation of blackberry juice and *Lactobacillus acidophilus* by gum arabic–maltodextrin could be effective to protect anthocyanins and probiotic bacteria (63). In addition, alginate–pectin hydrogel particles have been reported to encapsulate blueberry anthocyanins with high encapsulation efficiency (116%) (64).

### Combination of Proteins and Polysaccharides for Encapsulation

Covalent interaction is the main pathway that contributes to the interactions between proteins and polysaccharides. Several factors affect covalent interactions, such as intrinsic factors, including free amino groups, carbonyl groups, molecular structure, hydrophilicity, and hydrophobicity. Similarly, extrinsic factors, such as pressure, temperature, processing methods (i.e., microwave, ultrasonic, and pulsed electric field), crosslinkers, and the molar ratio between biopolymers, affect the interactions between proteins and polysaccharides.

The covalent bonds formed by proteins and polysaccharides are involved in the enhancement of the stability and impediment of anthocyanin release in harsh environments (65). During this process, polysaccharides and proteins or peptides form electrostatic complexes by opposite charges under particular pH conditions. The covalent bonds can be achieved via chemical cross-linking or Maillard reactions. Anthocyanins interact with proteins via hydrophobic interactions and hydrogen bonds because of the high affinity between anthocyanins and proteins (13, 66). Afterward, the loaded proteins can be cross-linked by electrostatic interaction with oppositely charged polysaccharides to form double polymers (67, 68).

Electrostatic interactions between differently charged acrosome molecules lead to the formation of protein–polysaccharide complexes. This technique consists of two parts: the phase separation of biopolymer mixtures and the subsequent deposition of a cohesive phase near the active ingredients (69, 70). Three main steps, namely, the solubilization of biopolymers, mixing the biopolymers with appropriate proportions, and the acidification of the medium, are required to form complexes. Moreover, the acidification phase is critical because it strongly affects the complex dimensions of formation (32).

The biopolymers formed by proteins or peptides and polysaccharides are promising for anthocyanin encapsulation because they could achieve high loading capacity and encapsulation efficiency and controlled release (71). Whey protein, gum arabic, and maltodextrin have been employed for anthocyanin extract encapsulation using freeze drying with encapsulation efficiency over 82%; they could reduce anthocyanin degradation during heat processing (72). Moreover, the biopolymer particles fabricated with beet pectin and whey protein have been used to encapsulate anthocyanins to improve their heat stability (31). Anthocyanins from elderberry were encapsulated through whey proteins and pectin with high encapsulating efficiency (98%), and the remarkable anti-oxidation of the system highlighted the potential utilization of the microcapsules in food products (41).

As shown, the biopolymers of proteins and polysaccharides for anthocyanin encapsulation can be formed by covalent interactions and non-covalent complexations, and the possible factors that might be involved in the formation are summarized in previous studies (73). In comparison with the anthocyanin encapsulation based on proteins or polysaccharides, the protein-polysaccharide systems for anthocyanin encapsulation are comparable or more excellent for the improvement of stability in harsh environments and may overcome the limitation of single utilization (9, 74).

**Figure 5:**
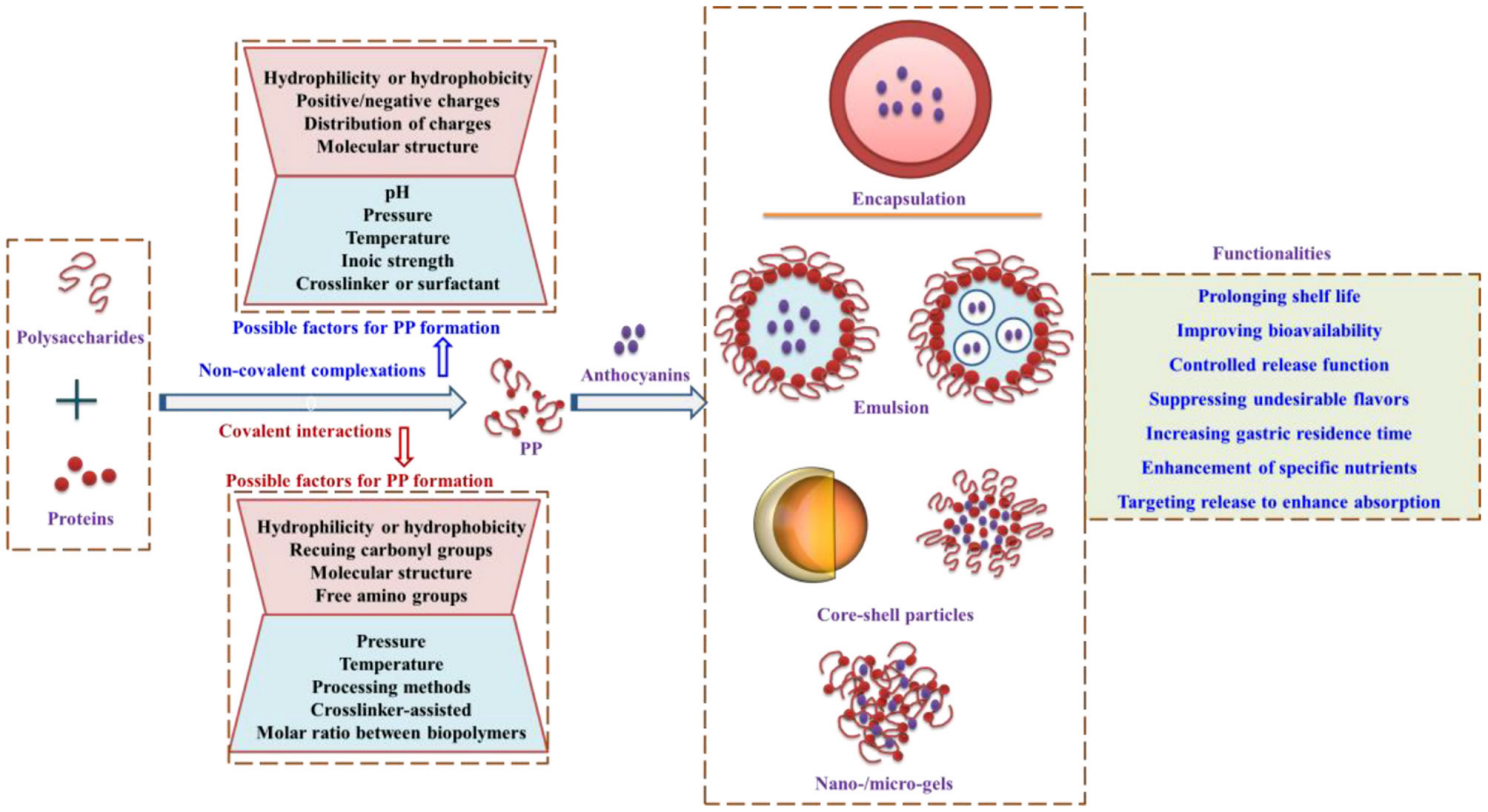
Biopolymers formed by proteins and polysaccharides for anthocyanin encapsulation, the possible factors that affect biopolymer formation, and the functionality of encapsulation. PP, Protein–polysaccharides. Adapted from reference (57, 73, 76–78) with permission.

The strategy of anthocyanin encapsulation has presented functionalities in improving stability, increasing gastric residence time, and targeting release to enhance anthocyanin uptake and absorption by the formation of nanogels, microgels, microparticles, or emulsion systems (17, 75). The protein- and polysaccharide-based biopolymers for anthocyanin encapsulation (Figure 5) provide new insights for further research on how to protect anthocyanins against the external harsh environment by the utilization of environmentally friendly biopolymers.

### Interactions Between Proteins and/or Polysaccharides and Anthocyanins

The absorption and excretion of anthocyanins are associated with many factors; among which, the food matrix's effects are important to maintain the bioactivities of dietary anthocyanins (79, 80). As important parameters, the non-covalent interactions of anthocyanins with proteins, and/or carbohydrates have attracted intensive research attention (81). These interactions with macronutrients, which are driven by van der Waals interactions, hydrogen bond, and hydrophobic interaction, could affect anthocyanins' properties, including bioavailability and radical scavenging (82).

Anthocyanin–protein complexes can be formed by crosslinking or aggregation via non-covalent binding. The hydroxyl and terminal galloyl groups of anthocyanins may contribute to the modulation of crosslinking owing to their molecular flexibility (82, 83). Moreover, anthocyanin–protein (non-enzyme) interactions may also be involved in subtle conformational changes (84). Non-covalent interactions may also occur between anthocyanins and carbohydrates (Figure 2). Generally, the consumption of plant anthocyanins involves the ingestion of starch and fibers, which may help improve their stability by counteracting the pH variations in different *in vivo* digestion phases (85). The physical entrapping induced by these molecules restricts the mixing process between digestive fluids and anthocyanins to avoid their degradation to some extent and facilitate the biomolecules to reach the gut wall, which can improve their bioavailability and health-promotion benefits (12, 34).

Proteins, polysaccharides, and other components in the food matrix are commonly worked together to affect anthocyanin or macronutrient digestion. All ingredients work together to produce a final result, which highlights that the effects of the food matrix should be evaluated by taking into account all the ingredients or at least the main contributors. The observed effects and interactions of the matrix with anthocyanins remain elusive and require further investigation (77, 78, 86).

## Current Understanding and Future Perspectives

The non-targeted release and low stability are the major obstacles of anthocyanins to present health benefits in food systems (87–89). Recently, encapsulation approaches have been developed to address the low stability, low oral bioavailability, and poor intestinal absorption of anthocyanins. Several emerging micro/nanoencapsulation approaches are effective to some extent for improving anthocyanins' stability against the harsh environment of the gastrointestinal tract with bio-efficacy enhancement (90, 91). In encapsulation, particle aggregation and particle size control, the sensitivity to pH and ionic strength of the prepared particles, as well as other related factors, should be optimized for the practice applications with satisfactory stability and bioavailability (92, 93). As above, the application of emerging micro/nanoencapsulation techniques in the food industry is still challenging.

**Figure 6:**
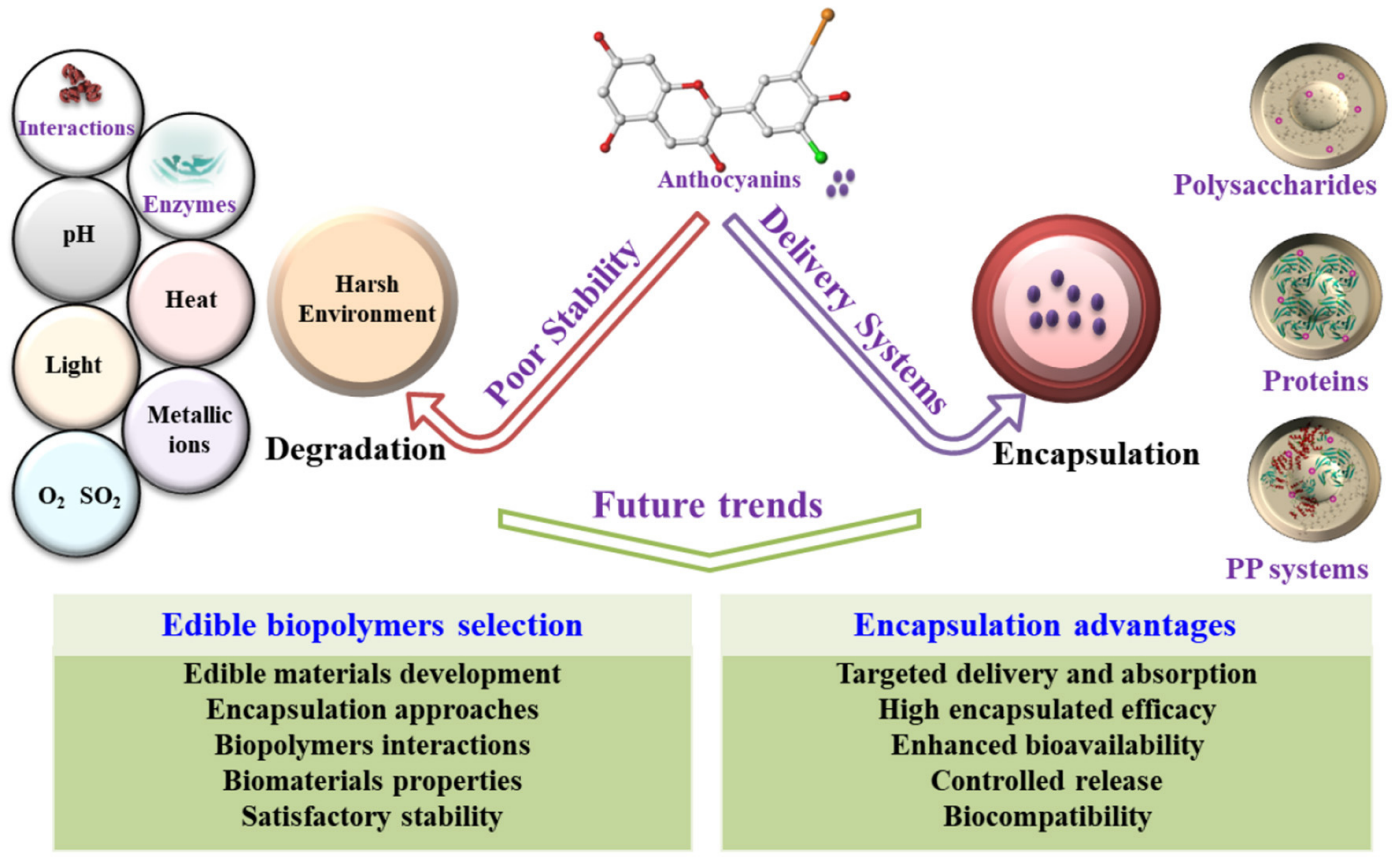
Framework of the future trends or advantages of the micro-/nanoencapsulation of anthocyanins using edible biopolymers, including proteins or/and polysaccharides. PP, protein-polysaccharide.

Only food-grade biomaterials can be employed and accepted for delivering anthocyanins in the food industry. Regardless of nano/microcapsulation technique, food-grade materials, such as proteins, and polysaccharides, are utilized as wall materials for anthocyanin encapsulation with the promising performance of high encapsulated efficacy, enhanced stability, and excellent biocompatibility. The interactions between anthocyanins (e.g., proteins/peptides and polysaccharides) and biomaterials are important in designing delivery systems (Figure 6). The biomaterials properties, satisfactory stability, and the interactions between the biopolymers and anthocyanins should be considered when the edible biopolymers were selected for anthocyanin encapsulation. Although each method has advantages for specific applications, evaluating the requirements according to the advantages and disadvantages of encapsulation approaches is neccessary before selection.

Bioderived colloidal particles, including protein–polysaccharide conjugates, micro/nanogels, and microfibers, provide new insights into the development of biopolymer interfaces to replace emulsifier layers (94). The potential of stabilized interface for particles has attracted great attention for food colloidal structure research (95). Complex coacervation, which has received a growing interest, presents excellent loading capacity, mild operating conditions, and controlled release (96). These controlled parameters for polysaccharide–protein complexes can enhance functional properties without enzymatic and chemical modifications and support the excellent encapsulation of anthocyanins.

Future recommendations include the utilization of microencapsulated anthocyanins with satisfactory bioavailability and stability as food fortification components (97). Developing more biopolymers with health benefits as wall materials is also crucial. New edible biomaterials or the new combinations of known biomaterials for the effective microencapsulation or nanoencapsulation of anthocyanins are important for the satisfactory design of micro/nanomaterials with novel characteristics (98). In particular, research interest on the microcapsules of anthocyanins and other polyphenols for biologically triggering their release in living cells is increasing (99, 100). Additionally, further research is still suggested to combine the feasibility of different anthocyanin encapsulation techniques. However, seeking and strengthening the optimal techniques combined with environmental protection, high yield, and low cost are still needed.

The booming food industry will no longer be regarded as a low-profit commodity and will be a source of well-being and a revenue potential. The utilization of functional biopolymers via edible materials for food structure design provides new insights into the development of future foods with excellent sensory properties and health benefits, avoiding synthetic additives and negative nutrients. Importantly, investigating new edible biomaterials or creating new colloidal structures with underutilized edible biopolymers for future food design is an exciting and promising research direction.

## Author Contributions

JS, YY, ZL, and ZM designed the topic. JS and YY prepared the manuscript. JS, ZR, and ZL prepared the figures. YY, ZM, MC, ZR, LC, and CF reviewed and revised the manuscript. All authors contributed to the article and approved the submitted version.

## Conflict of Interest

The authors declare that the research was conducted in the absence of any commercial or financial relationships that could be construed as a potential conflict of interest.

## Publisher's Note

All claims expressed in this article are solely those of the authors and do not necessarily represent those of their affiliated organizations, or those of the publisher, the editors and the reviewers. Any product that may be evaluated in this article, or claim that may be made by its manufacturer, is not guaranteed or endorsed by the publisher.
